# Pros and cons of alternative therapy omega-3 fatty acids during pregnancy and lactation for mental problems

**DOI:** 10.1192/j.eurpsy.2024.1674

**Published:** 2024-08-27

**Authors:** L. Boronin, I. Nastas

**Affiliations:** Department of Mental Health, Medical Psychology and Psychotherapy, State University of Medicine and Pharmacy, Chisinau, Moldova, Republic of

## Abstract

**Introduction:**

Fatty acids omega-3 are irreplaceable. They stabilize cell membranes, nerve impulses, homeostasis, immune reactions, the birth process, the psycho-emotional state of the fetus-mother dyad. Correlations between adequate dietary intake of omega-3 and cognitive health have been described in detail. According to the literature, docosahexaenoic acid is associated with the synthesis of serotonin, dopamine, acetylcholine, glutamate, neuroprotective and anti-apoptotic action, has antidepressant effect. Omega-3 makes up 60% of neuronal membrane phospholipids. Under clinical aspect,according to publications, fish oil reduces the risk of preterm birth by 44%.

**Objectives:**

The aim is to study the risks and benefits of using omega-3 during pregnancy and lactation in patients with mental disorders.

**Methods:**

Comparative analysis of evidence-based scientific publications for the use of omega-3 fatty acids in pregnancy and lactation.

**Results:**

The body level of omega-3 depends on the quantitative intake from food, as well as gene polymorphism and age. For pregnant and lactating women are recommended 200-300 mg per day or about 300 g per week from food. Deficiency of omega-3 (protectins) affects the processes of myelination, neurogenesis, synaptogenesis, the metabolism of neurotransmitters, cell differentiation, neuronal migration and inflammatory responses.

**Conclusions:**

There are many probably mechanisms of action of omega-3, namely: Enhances peroxisomal oxidation, reduces the synthesis of triglycerides in the liver; inhibits plasma acyltransfrerase. Omega-3 acts on phospholipids of the cell membranes of the nervous system and retina, their adequate functioning, improve psychomotor development of newborns. It was found the effect of decreasing the levels of cytokines and depressive symptoms, as well the risk of food allergies and depression. In conclusion, in adequate doses, omega-3 fatty acids seems to be useful in deficiencies and for prophylactic purposes in pregnancy and lactation.

**Keywords:**

omega- 3 fatty acids, pregnancy, lactation, mental disorders.

**Disclosure of Interest:**

None Declared

